# Variations in genetic diversity in cultivated *Pistacia chinensis*

**DOI:** 10.3389/fpls.2022.1030647

**Published:** 2022-11-10

**Authors:** Biao Han, Ming-Jia Zhang, Yang Xian, Hui Xu, Cheng-Cheng Cui, Dan Liu, Lei Wang, De-Zhu Li, Wen-Qing Li, Xiao-Man Xie

**Affiliations:** ^1^ Key Laboratory of State Forestry and Grassland Administration Conservation and Utilization of Warm Temperate Zone Forest and Grass Germplasm Resources, Shandong Provincial Center of Forest and Grass Germplasm Resources, Ji’nan, Shandong, China; ^2^ College of Forestry, Shandong Agricultural University, Tai’an, Shandong, China; ^3^ Germplasm Bank of Wild Species, Kunming Institute of Botany, Chinese Academy of Sciences, Kunming, Yunnan, China

**Keywords:** discordance, genetic diversity, nuclear SNPs, *Pistacia chinensis*, plastome

## Abstract

Identification of the evolution history and genetic diversity of a species is important in the utilization of novel genetic variation in this species, as well as for its conservation. *Pistacia chinensis* is an important biodiesel tree crop in China, due to the high oil content of its fruit. The aim of this study was to uncover the genetic structure of *P. chinensis* and to investigate the influence of intraspecific gene flow on the process of domestication and the diversification of varieties. We investigated the genetic structure of *P. chinensis*, as well as evolution and introgression in the subpopulations, through analysis of the plastid and nuclear genomes of 39 P*. chinensis* individuals from across China. High levels of variation were detected in the *P. chinensis* plastome, and 460 intraspecific polymorphic sites, 104 indels and three small inversions were identified. Phylogenetic analysis and population structure using the plastome dataset supported five clades of *P. chinensis*. Population structure analysis based on the nuclear SNPs showed two groups, clearly clustered together, and more than a third of the total individuals were classified as hybrids. Discordance between the plastid and nuclear genomes suggested that hybridization events may have occurred between highly divergent samples in the *P. chinensis* subclades. Most of the species in the *P. chinensis* subclade diverged between the late Miocene and the mid-Pliocene. The processes of domestication and cultivation have decreased the genetic diversity of *P. chinensis.* The extensive variability and structuring of the *P. chinensis* plastid together with the nuclear genomic variation detected in this study suggests that much unexploited genetic diversity is available for improvement in this recently domesticated species.

## Introduction

The genus *Pistacia* (Anacardiaceae) consists of at least 11 species (Parfitt and Badenes, 1997; Xie et al., 2014). The species are trees or shrubs and are dioecious, with female and male flowers on separate trees, and the fruit is a monocarpic drupe. Chinese pistache tree (*Pistacia chinensis* Bunge) is a small, wind-pollinated tree species with apetalous flowers, and is widely distributed throughout China owing to its strong adaptability to poor habitat and adverse conditions. This tree has potential as a biodiesel tree species in China due to the high oil content of its fruit (Li et al., 2010). The oil content in the seed is typically higher than 40% and the sixteen alkyl value of biodiesel derived from the seeds is generally up to 51.3 (Wang et al., 2012). *Pistacia chinensis* has been used as a landscape tree and as a vegetable, and is also used as rootstock for *P. vera*, because it is strongly adaptable and resistant to adverse conditions (Tang et al., 2012). Additionally, *P. chinensis* is also used in Chinese traditional medicine to relieve dysentery, inflammatory swelling, psoriasis and rheumatism (Tang et al., 2012).

Deterministic or stochastic forces, such as domestication or genetic drift, may decrease genetic diversity at different levels of biological organization, for example at the individual, population or species level. Evolutionary forces such as dispersal, hybridization or introgression can lead to decreases or increases in divergence among difference subpopulations, obscuring the origins of domestication and mixing the genetic variations. *P. chinensis* is a recently domesticated species, and in this important biodiesel tree species, understanding the genetic diversity of the wild germplasm is essential in order to prioritize conservation of novel wild germplasm that may be useful in the future, and to guide the introduction of novel genetic diversity into selective breeding populations.

Genetic diversity in *P. chinensis* has previously been studied using several markers, including SSR (Wu et al., 2010; Lu et al., 2019; Cheng et al., 2022), random amplified microsatellite polymorphism (RAMP), random amplified polymorphic DNA (RAPD) and amplified fragment length polymorphism (AFLP) (Katsiotis et al., 2003). Parfitt and Badenes and Xie et al. (Parfitt and Badenes, 1997; Xie et al., 2014) used plastid and nuclear genome sequences in phylogenetic and biogeographic analyses of *Pistacia*, however, to date, few studies, if any, have examined extensive genomic SNP data (including the plastid and nuclear genomes) for population analyses. The advantages of DNA sequence markers include their high reproducibility, increasing the chances and the abilities of detecting genetic diversity (Kumar et al., 2009).

With advances in sequencing methods, genomic data in particular are a popular in the evaluation of population genetics (Fu et al., 2022; Karbstein et al., 2022). This has led to scientists focusing on the nuclear genome and paying little attention to the plastome, which is considered to have lower divergence within species. However, recently, certain evolutionary studies have been conducted at the intraspecies level based on plastomes, providing further insight into the biogeographical structure and extensive genetic variation at the population level (Perdereau et al., 2017; Magdy et al., 2019; Migliore et al., 2019; Mohamoud et al., 2019). Plastids, with their highly conserved maternally inherited genomes, show a clear geographical structure (Hohmann et al., 2018; Xue et al., 2021), and are therefore useful in phylogeographical studies. Therefore, combining plastid and nuclear genome sequences allows us to conduct comprehensive investigations into genetic diversity because the genetic information available is maternally and biparentally inherited, respectively.

In this study, we sequenced the genomes of 39 P*. chinensis* individuals from across China. Whole plastomes and nuclear SNPs were assembled and analyzed, and we then used this huge genetic variation to characterize the structure and diversity of *P. chinensis* from the differences among the individuals. We then compared the plastid and nuclear genomes and investigated possible gene flow and introgression occurring during the domestication of *P. chinensis*. Our results not only reveal evolutionary factors responsible for reshaping the genetic variation in *P. chinensis* populations, but also exemplify changes in genetic diversity during the domestication and cultivation processes.

## Materials and methods

### Sampling and DNA extraction

We collected a total of 39 samples of *P. chinensis* from across China and deposited them in the Shandong Provincial Center of Forest Tree Germplasm Resources, Jinan, China (**Table 1**). Due to the widespread cultivation of *Pistacia chinensis*, we conducted random sampling of accessions from the same area. The sampled accessions were all collected from within the natural range of this species. Fresh leaf material was dried in silica gel, and voucher specimens were deposited in the herbarium of the Shandong Provincial Center of Forest Tree Germplasm Resources. About 0.02 g of dried leaf tissue was ground using a mechanical lapping method, and total genomic DNA was extracted using a modified CTAB protocol (Li et al., 2013). DNA concentration was quantified using a Qubit 2.0 Fluorometer (Thermo Fisher Scientific, Inc., Carlsbad, CA, USA), and the size and quality of the DNA were visualized and assessed using a TAE agarose gel.

**Table 1:** Sample information and the size of the chloroplast genome sequences.

Voucher	Samples	Location	LSC	IR	SSC	Total length	Genbank accession number
BJFC 00021725	Anhui	Huaning County, Huangshan City, Anhui Province	88371	26595	19057	160618	OP554543
BJTZS	Beijing	Beijing City	88371	26595	19057	160618	OP554540
202109ZPF0133	Gansu	Hui County, Longnan City, Gansu Province	88371	26595	19057	160618	OP554537
BJFC 00021710	Guangxi	Nanning City, Guangxi Province	88526	26595	19090	160806	OP554555
BJFC 00076565	Guizhou01	Congjiang County, Qiandongnan, Guizhou Province	88371	26595	19057	160618	OP554538
BJFC 00078597	Guizhou02	Rongjiang County, Qiandongnan, Guizhou Province	88366	26595	19087	160643	OP554557
202009zlj069	Hebei01	Wuan City, Handan, Hebei Province	88371	26595	19057	160618	OP554536
202009zlj070	Hebei02	Wuan City, Handan, Hebei Province	88371	26595	19057	160618	OP554535
BJFC 00081493	Henan01	Xinxiang City, Henan Province	88371	26595	19057	160618	OP554541
BJFC 00081484	Henan02	Xinxiang City, Henan Province	88387	26596	19088	160667	OP554550
BJFC 00079805	Henan03	Xinxiang City, Henan Province	88387	26596	19088	160667	OP554549
BJFC 00085509	Henan04	Xinxiang City, Henan Province	88388	26596	19088	160668	OP554552
BJFC 00100722	Jiangsu01	Nanajing City, Jiangsu Province	88371	26595	19057	160618	OP554539
202009bzg003	Jiangsu02	Haizhou District, Lianyungang City, Jiangsu Province	88371	26595	19057	160618	OP554534
202009bzg020	Jiangsu03	Haizhou District, Lianyungang City, Jiangsu Province	88371	26595	19057	160618	OP554533
202009bzg083	Jiangsu04	Lianyun District, Lianyungang City, Jiangsu Province	88546	26595	19085	160821	OP554553
202009bzg154	Jiangsu05	Pukou District, Nanjing City, Jiangsu Province	88371	26595	19057	160618	OP554532
BJFC 00021716	Jiangxi	Xunwu County, Ganzhou City, Jiangxi Province	88546	26595	19084	160820	OP554554
201909wfs049	Shandong01	Muping District, Yantai City, Shandong Province	88371	26595	19057	160618	OP554531
201910lq011	Shandong02	Changqing District, Jinan City, Shandong Province	88387	26596	19087	160666	OP554551
201910lw012	Shandong03	Huangdao District, Qingdao City, Shandong Province	88371	26595	19057	160618	OP554529
201910lw014	Shandong04	Huangdao District, Qingdao City, Shandong Province	88371	26595	19057	160618	OP554542
201910lw016	Shandong05	Huangdao District, Qingdao City, Shandong Province	88371	26595	19057	160618	OP554528
201910lw017	Shandong06	Huangdao District, Qingdao City, Shandong Province	88371	26595	19057	160618	OP554527
201910lw005	Shandong07	Huangdao District, Qingdao City, Shandong Province	88371	26595	19057	160618	OP554530
202009hb040	Shandong08	Tai’an City, Shandong Province	88387	26596	19088	160667	OP554548
202009gxj042	Shandong09	Tantai City, Shandong Province	88371	26595	19057	160618	OP554526
202009wl165	Shanxi01	Mei County, Baoji City, Shaanxi Province	88371	26595	19057	160618	OP554525
202110040028	Shanxi02	Feng County, Baoji City, Shaanxi Province	88371	26595	19057	160618	OP554521
202110170077	Shanxi03	Feng County, Baoji City, Shaanxi Province	88371	26595	19057	160618	OP554520
202110040060	Shanxi04	Feng County, Baoji City, Shaanxi Province	88405	26596	19089	160686	OP554544
202110040075	Shanxi05	Feng County, Baoji City, Shaanxi Province	88371	26595	19057	160618	OP554522
202109WL0154	Shanxi06	Loyang County, Hanzhong City, Shaanxi Province	88405	26596	19089	160686	OP554545
202109WL0099	Shanxi07	Loyang County, Hanzhong City, Shaanxi Province	88371	26595	19057	160618	OP554523
202109WL0093	Shanxi08	Loyang County, Hanzhong City, Shaanxi Province	88405	26596	19089	160686	OP554546
202109WL0064	Shanxi09	Loyang County, Hanzhong City, Shaanxi Province	88405	26596	19089	160686	OP554547
202009wl251	Shanxi10	Zhouzhi County, Xi’an City, Shaanxi Province	88371	26595	19057	160618	OP554524
BJFC 00021739	Yunnan01	Kunming City, Yunan Province	88481	26595	19083	160754	OP554556
202110110137	Zhenjiang	Lin’an District, Hangzhou, Zhejiang Province	88371	26595	19057	160618	OP554519

### Library preparation and sequencing

A total of 700 ng DNA per sample was used for library preparation. Genomic DNA was fragmented by sonication into 350 bp fragments. Sequencing libraries were generated using NEB Next^®^ Ultra™ DNA Library Prep Kit for Illumina (NEB, USA) and was then used for sequencing. Each sample was barcoded with a unique index, and libraries were pooled. Whole-genome shotgun sequence data was paired-end sequenced (2 × 150 bp) on an Illumina HiSeq X-ten platform (Illumina, Inc., San Diego, CA, USA). Most samples yielded approximately 15 Gb of 150-bp paired-end reads, which is about 30 X depth of coverage for the genome of *Pistacia chinensis*.

### Assembly and annotation of the plastome

Quality control of raw reads was conducted using Trimmomatic version 0.39 (Bolger et al., 2014) with the following options: LEADING, 20; TRAILING, 20; SLIDING WINDOW, 4:15; MIN LEN, 36; and AVG QUAL, 20. Clean reads were used to assemble the plastome of *P. chinensis* using GetOrganelle, with a range of k-mers of 75, 85, 95, and 105 (Jin et al., 2020). Where GetOrganelle failed to assemble the complete plastome, we assembled it following the methods of Dong et al. (2022). Gene annotation of the plastome was performed with Plann (Huang and Cronk, 2015), and the published genome of *P. chinensis* (GenBank accession number: MT157378) was used as the reference sequence. The physical map of the *P. chinensis* plastome was drawn in Chloroplot (Zheng et al., 2020).

### Analysis of variation in the plastome

The genome sequences from the 39 P*. chinensis* individuals were aligned using MAFFT version 7.490 (Katoh and Standley, 2013) and adjusted manually using Se-Al version 2.0 (Rambaut, 1996) to avoid alignment errors, such as polymeric repeat structures and small inversions. Nucleotide diversity, number of indels and sequence distance were used to assess sequence divergence over all the plastomes. The number of variable sites, parsimony-informative sites and sequence distances (π) were calculated using MEGA version 7.0 (Kumar et al., 2016). Nucleotide diversity and number of indels were calculated using DnaSP version 6 (Rozas et al., 2017).

### Reference mapping and nuclear SNP calling

Clean reads were mapped to the pistachio (*Pistacia vera*) reference genome (Zeng et al., 2019) using the program BWA version 0.7.17 (Li and Durbin, 2010) with default settings. Potential PCR duplicates were removed using SAMtools version 1.3.1 (Li et al., 2009). Only uniquely mapped paired reads were used for the detection of SNPs. The high-quality nuclear SNPs were called through GATK version 4.2.0.0 (Heldenbrand et al., 2019) and Picard tools version 1.92 (http://broadinstitute.github.io/picard/). The SNPs were then extracted and filtered according to the following criteria: quality value ≥ 20; sites with coverage over 2; missing data less then 10%. The SNP VCF files were then merged together with VCFtools version 0.1.14.

### Phylogenetic analysis of *Pistacia chinensis* individuals

The plastome sequences of the 39 sampled *P. chinensis* individuals, with that of *P. weinmaniifolia* as the outgroup, were aligned using MAFFT version 7.490 (Katoh and Standley, 2013). A phylogenetic tree based on this plastome dataset was then reconstructed using a maximum likelihood (ML) method in RAxML-NG (Kozlov et al., 2019). The best-fit model for ML analysis was found to be ModelFinder (Kalyaanamoorthy et al., 2017) based on Bayesian information criteria.

*P. vera* was used as the outgroup for the nuclear SNPs dataset. In order to investigate intraspecific hybridization among the individuals, we used the following dividing method to infer the phylogenetic relationships within the nuclear SNPs dataset, thereby avoiding concatenation-based ML analyses. In this method, each 100 kb of SNPs were divided into a new data matrix and used for tree reconstruction. ML trees were inferred using IQ-TREE version 2 (Minh et al., 2020) and branch support values were computed using the UFBoot method.

### Population structure and PCA analysis

The plastome and nuclear SNPs datasets were used to examine the population ancestry. ADMIXTURE was used to investigate the population genetic structure of all individuals, specifying K values ranging from 1 to 10 (Alexander and Lange, 2011). The optimum number of clusters (K) was determined at the K value with the lowest cross-validation error. Principal component analysis (PCA) was also conducted to evaluate the genetic structure of *P. chinensis* using Plink (Purcell et al., 2007), and graphs were built using the ggbiplot package in R. We constructed a network using the plastome dataset. Haplotype data were analyzed in DnaSP version 6 (Rozas et al., 2017) and a TCS network was built using PopArt version 1.7 (Clement et al., 2002; Leigh and Bryant, 2015).

### Analysis of intraspecific hybridization

We divided all samples into three populations with multiple individuals according to the Admixture result K = 2. TreeMix version 1.12 (Pickrell and Pritchard, 2012) was used to estimate gene flows between different populations, with blocks of 200 SNPs to account for linkage disequilibrium, and standard errors of migration rates were also calculated. The outputs were visualized in R.

### Estimation and profiling of divergence time

We used the complete plastome to estimate the divergence times of the different haplotypes. This dataset included 12 haplotypes of *P. chinensis* and a further 28 species from the Anacardiaceae. Four priors were used for this analysis. The root age of the tree (crown age of Anacardiaceae) was set to 70 Ma according to the wood fossils related to the Anacardiaceae and Burseraceae, which were reported from the upper Cretaceous of Mexico. The minimum stem age of *Rhus* was calibrated as 44 Ma, according to the fruit fossils of *Rhus*, from the middle Eocene of western North America (Manchester, 1994). The other two priors were taken from the findings of Xie et al. (Xie et al., 2014): the crown age of *Rhus* was set as 33.24 Ma and the stem age of *Cotinus* was set to 37.6 Ma.

BEAST 2 (Bouckaert et al., 2014) was used to perform the divergence time analyses. A GTR model and an uncorrelated lognormal distribution relaxed molecular clock model were selected. A Markov Chain Monte Carlo (MCMC) algorithm was run for 500,000,000 generations, sampling every 50,000 generations. Convergence was assessed using Tracer version 1.6 (Rambaut et al., 2014) with effective sampling sizes (ESS) in all parameters surpassing 200. The first 10% of the trees were discarded as burn-in and the remaining trees were used to construct the Maximum Clade Credibility (MCC) tree with mean heights in TreeAnnotator.

## Results

### The *Pistacia chinensis* plastome and sequence variation

**Figure 1:**
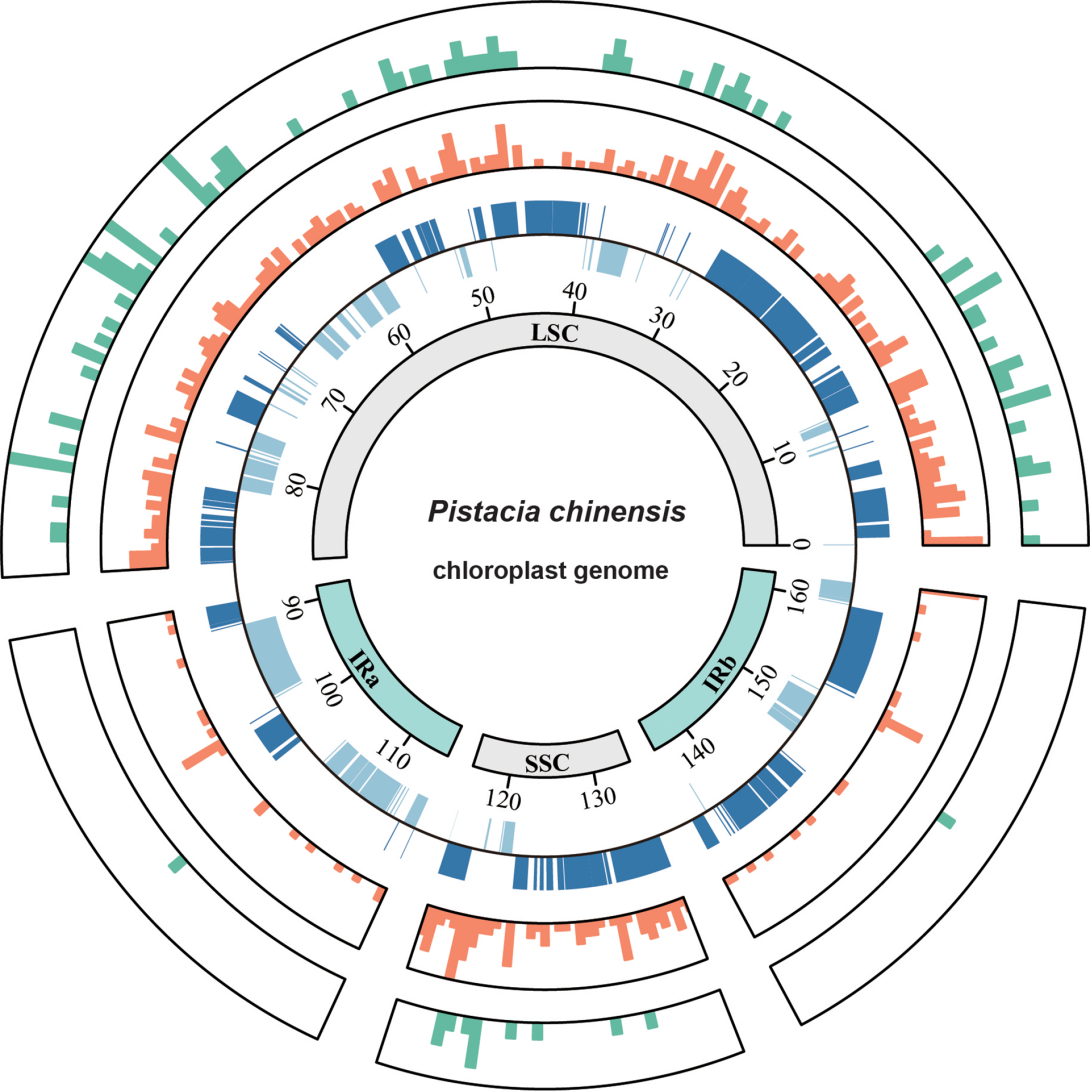
Circos plot showing the indel and nucleotide diversity of the *Pistacia chinensis* plastome. The concentric circles (inner to outer) indicate the following: quadripartite structure (represented by different colors); location of genes in the plastome; nucleotide diversity; and the number of indels. Nucleotide diversity and number of indels were computed for windows of 500 bp.

The plastome of *P. chinensis* ranged from 160,618 to 160,821 bp in length (**Table 1**) and consisted of four distinct parts, including a large single copy (LSC) region, a small single copy (SSC) region, and a pair of inverted repeats (IRA/IRB), exhibiting similar structure typical of most angiosperm species (**Figure 1** and **Figure S1**). The LSC (between 88,366 bp and 88,546 bp) and SSC (between 19,057 bp and 19,090 bp) were separated by the two IR regions (between 26,595 bp and 26,596 bp). The overall GC content was 37.9%, and the GC content was slightly higher in the IR (42.9%) regions than in the LSC (36.0%) and SSC (32.4%) regions. The annotated *P. chinensis* plastome included 113 unique genes (79 protein-coding genes, 30 tRNA genes, and four rRNA genes), with 60 protein-coding and 22 tRNA genes in the LSC, 11 protein-coding and one tRNA genes in the SSC, and with eight protein-coding genes, seven tRNAs and all four rRNAs in the IR region. Of these genes, 16 contained one intron and two (*clpP* and *ycf3*) contained two introns.

The alignment of the 39 P*. chinensis* plastomes was 161,388 bp in length, and included 460 variable sites, 104 indels and three small inversions. The overall genetic diversity was 0.00082. Most of intraspecific *P. chinensis* variable sites and indels were located in the LSC and SSC regions (**Figure 1**), indicating that the IR region was more conserved than the single copy regions. The average number of intraspecific variable sites was 2.9 per kb and the and indel density was 0.65 per kb. Nucleotide diversity averaged over 500 bp windows showed that two intergenic regions of *trnH-psbA* and *ndhF-rpl32* had the highest sequence divergence (**Figure 1**).

Of the 104 indels in the 39 P*. chinensis* plastomes,72 were SSR-related indels, 21 were repeat-related indels, and 11 were normal indels. All SSR-related indels were located in the non-coding regions. The indel size ranged from 1 to 9 bp, and 1 bp indels was present at the highest frequency (58.3%). Except for the indel in *clpP-psbB*, which was 3 bp long, all the normal indels were 1 bp long. The repeat-related indels ranged from 4 to 75 bp, with the longest occurring in the *ycf3-trnS* region, and found in an insert in the Yunnan01 sample.

All three small inversions formed stem-loop structures, and the lengths of these inversions were 3, 2, and 4 bp with the franking repeats of size 14, 22, and 14 bp, respectively. The lengths of the inversions and the flanking repeats were not correlated, which is consistent with previous research. The three small inversions were located in *atpF-atpH*, *petD-rpoA*, and *rpl14-rpl16*, and all of them occurred in the non-coding regions of the LSC.

### Genetic diversity and intraspecific differentiation based on the plastome dataset

**Figure 2:**
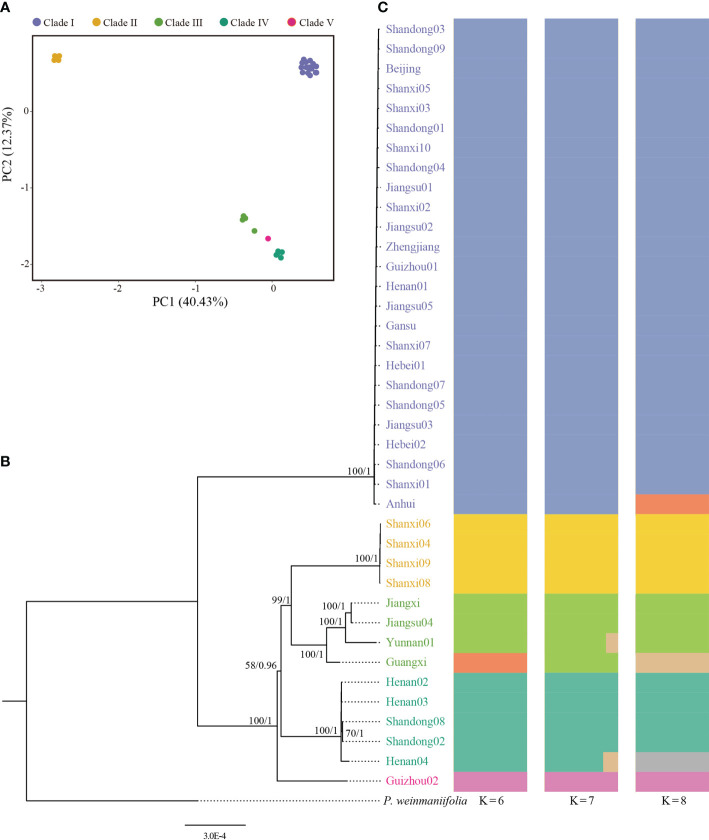
Genetic diversity of *Pistacia chinensis* assessed using complete plastome sequences. **(A)** Principal component analysis, **(B)** Phylogenetic tree. ML bootstrap support values/Bayesian posterior probabilities are shown at each node. **(C)** Population structure analysis with K = 6, 7 and 8.

We inferred phylogenetic trees using ML and BI methods, based on whole plastome sequences. All the 39 samples were clearly divided into five clades (**Figure 2B**). Population structure results from ADMIXTURE suggested that there were six clades with K=6 (**Figure 2C**, **Figure S2**). The PCA results revealed three major groups (**Figure 2A**).

**Figure 3:**
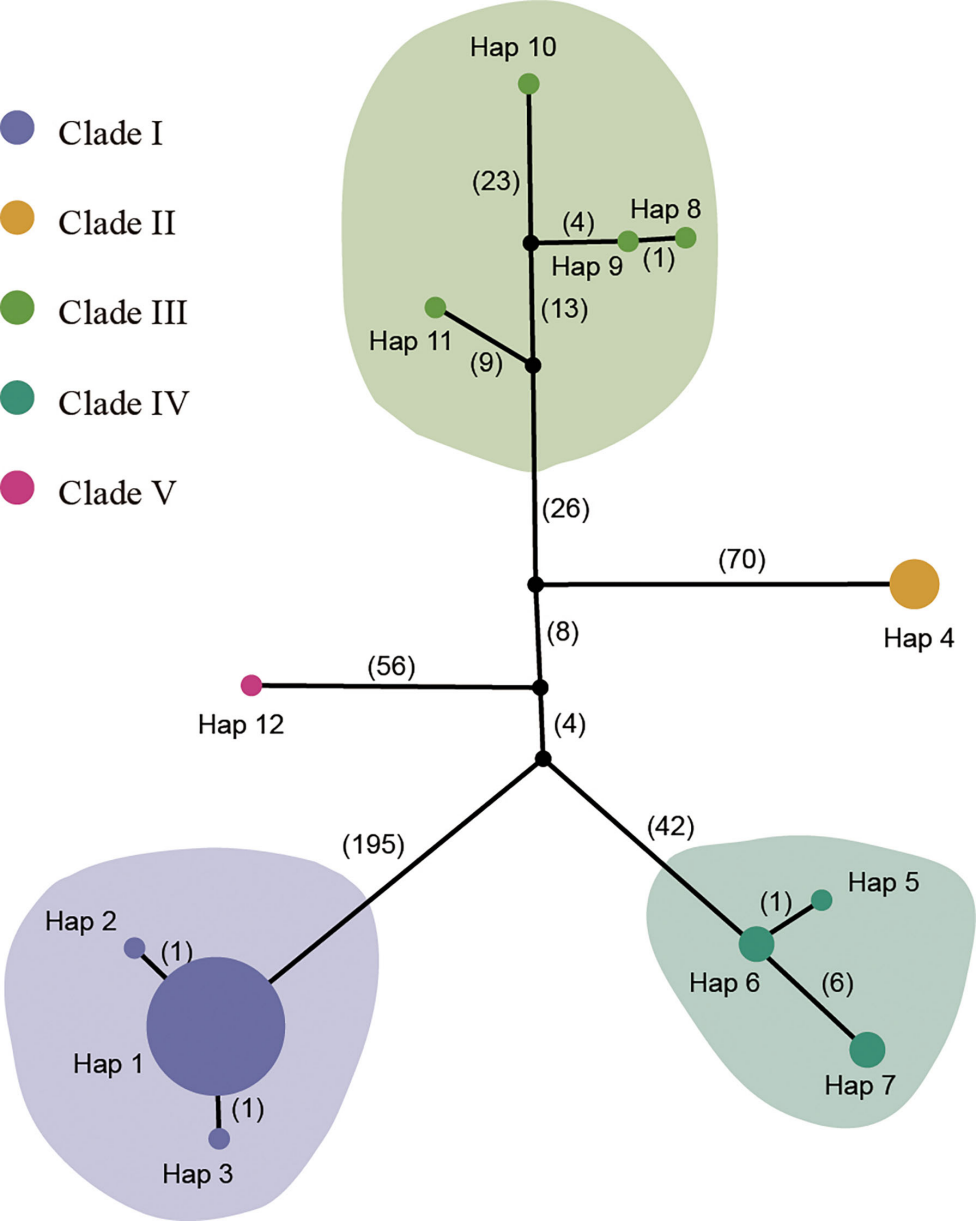
TCS network of 12 haplotypes from the plastome sequences. The colored circles represent plastid haplotypes; the black circles are extinct haplotypes; the number of mutational steps is shown on the lines. The size of the pie chart represents the number of the accessions. The haplotype for each sample is listed in **Table S1**.

The first principal component explained 40.43% of total variance and clearly separated Clade I and Clade II. In total, 12 distinct plastid haplotypes were identified, differing by between one and 279 plastid SNPs (**Figure 3**). The network of plastid haplotypes supported there were five clades.

The first clade contained 25 samples from Shandong, Beijing, Shanxi, Jiangsu, Zhejiang, Gansu, Guizhou, Hebei, and Anhui. This clade contained three haplotypes (Hap 1, Hap 2 and Hap 3), with Hap 1 being the most common. Clade I, which was sister to the other clades, exhibited significant genetic difference from the other clades and had the highest number of mutational steps (195). Clades II and III formed a single, highly supported (BS/PP=100/1) clade, and was sister to Clade IV which had lower supported (BS/PP=58/0.96) (**Figure 2B**). Clade II contained four samples from Shanxi with a single genotype (Hap 4). Clade III contained four samples from Jiangxi, Jiangsu, Yunnan, and Guangxi. The four samples showed significant divergence and included four different haplotypes (Hap 8-Hap 11). Clade IV contained five samples from Henan and Shandong. A single sample from Guizhou (Guizhou02, from Rongjiang County, Qiandongnan, Guizhou) formed Clade V.

### Genetic diversity and intraspecific differentiation based on the nuclear SNPs dataset

**Figure 4:**
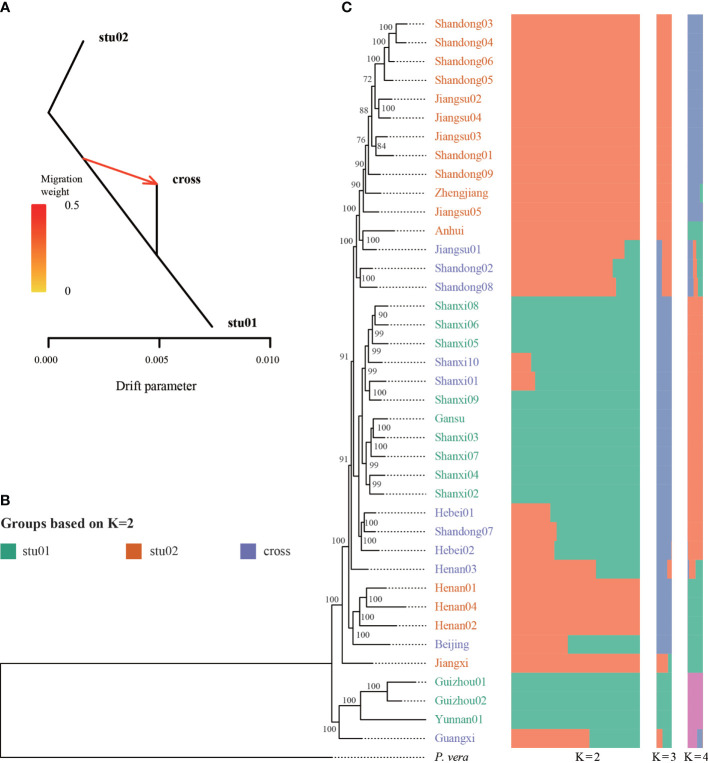
Genetic diversity of *Pistacia chinensis* based on nuclear SNPs. **(A)** Signal of introgression among different groups detected using the TreeMix program. According to the structure results (K = 2), we defined three groups, stu01, stu02 and the crossed (hybrid) samples. **(B)** Phylogenetic tree. ML bootstrap support values are shown at each node. **(C)** Population structure analysis with K = 2, 3 and 4.

Using the nuclear genome of *Pistacia vera* as the reference genome, we identified 3,632,308 SNPs with less than 10% missing data. The dataset of nuclear SNPs included only those sites that were polymorphic among the 39 sampled individuals. Population structure was analyzed using K values ranging from 1 to 10; the populations were clearly divided into two clades with K = 2, while the cross validation (CV) error was also the lowest with K = 2 (**Figure 4C**, **Figure S2**). The stu01 group contained nine samples from Jiangsu and Shandong. The stu02 group included 15 individuals from Gansu, Guangxi, Guizhou, Henan, Shanxi, and Yunnan. The remaining 15 individuals were classified as hybrids (the “cross” group), on the basis of the admixture coefficient according to the population structure at K = 2. The TreeMix results also identified strong gene flow from a node clustering stu01 and cross group into population cross (**Figure 4A**). The PCA results was showed in **Figure S3**.

Phylogenetic analysis of the 39 P*. chinensis* samples was performed based on the all the SNPs using the ML method (**Figure 4B**). Most of the nodes were well supported. Four samples (Guizou01, Guizhou02, Yunnan01, and Guangxi) formed a clade was the earliest diverged group. The two groups (stu01 and stu02) identified from ADMIXTURE did not form a monophyletic group.

### Discordance relationships between nuclear and plastomes

**Figure 5:**
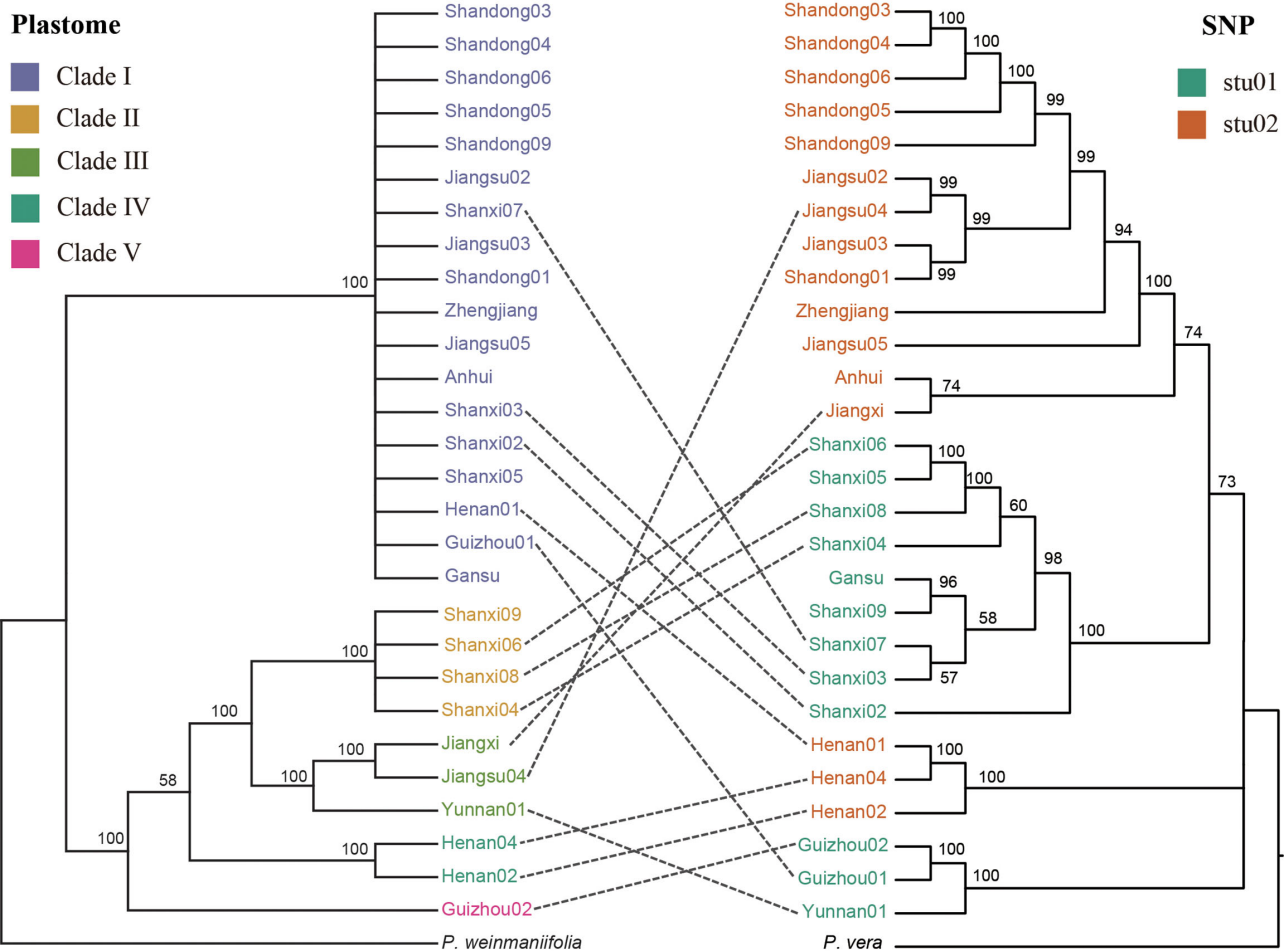
Discordance between phylogenetic trees reconstructed using plastomes (left) and nuclear SNPs (right). The trees included all the samples from the stu01 and stu02 clades from **Figure 4B**.

In order to discover the discordance relationships between nuclear and plastome dataset, we compared the two phylogenetic trees (**Figure 5**). The 15 samples were deleted in this analysis which were classified as hybrids according to the results of the population structure at K = 2. For the *P. chinensis* samples, more closely related samples according to their plastomes tend to share identical or more similar haplotypes of their nuclear genomes (**Figure 5**), suggesting co-evolution between the plastomes and the nuclear genomes as a general pattern. However, more apparent exceptions were also observed. For example, the two samples of Shanxi07 and Guizhou01 with highly diverged nuclear genomes were detected to share identical haplotype of the plastomes, and the two samples of Anhui and Jiangxi with more closely related nuclear genomes were detected to have more diverged haplotypes of the plastomes. The discordance phylogeny relationships between nuclear and plastomes suggest that hybridization events between highly diverged samples within the *P. chinensis* subclades have also occurred, and such events are likely to be responsible for the observed discordance between the nuclear and plastomes.

### Divergence time of *Pistacia chinensis*

**Figure 6:**
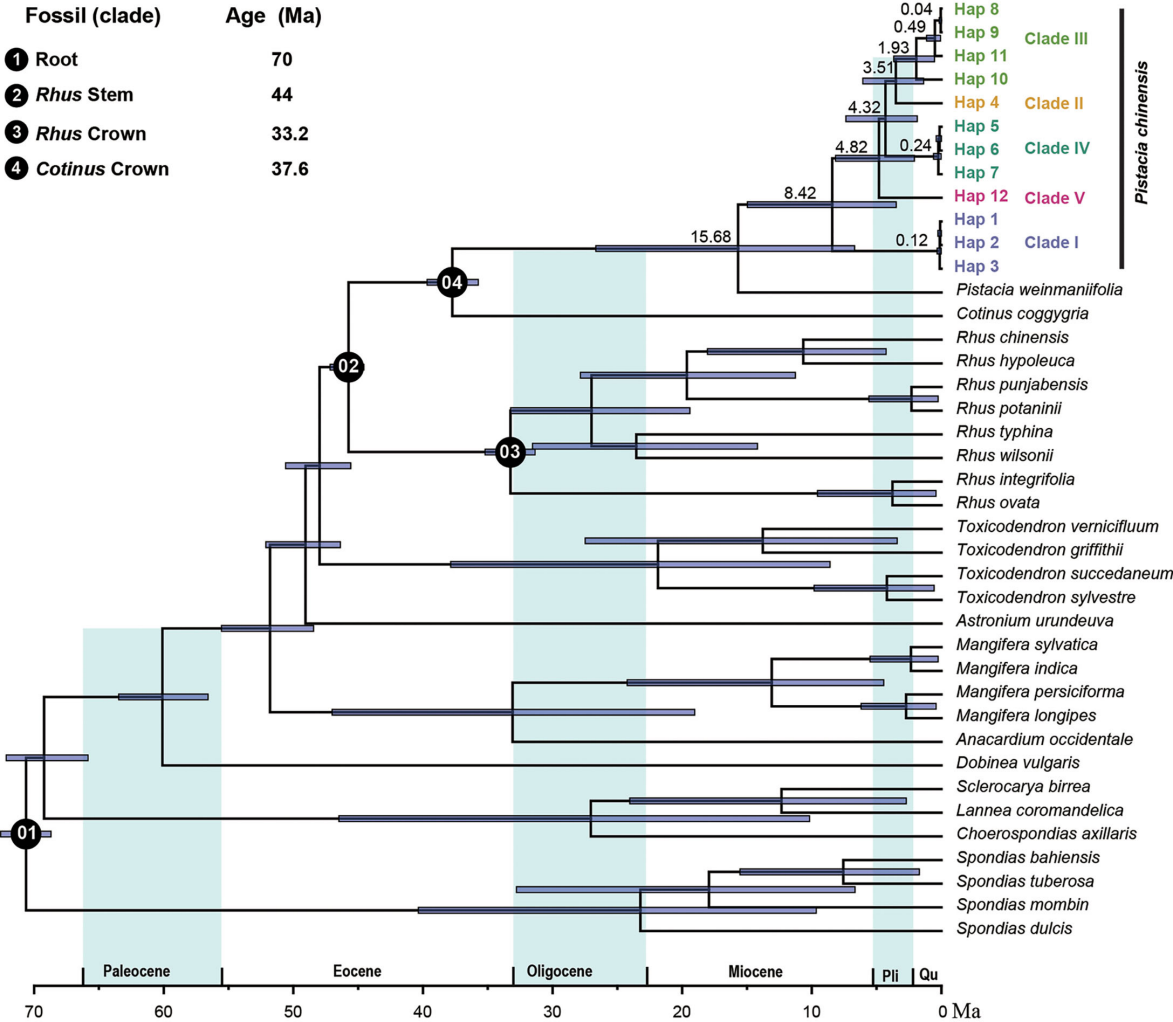
Divergence time of Anacardiaceae. The mean divergence time of the nodes is shown next to the nodes, and the blue bars correspond to the 95% highest posterior density (HPD).

Divergence time estimates showed that the stem and crown nodes of *Pistacia* were 37.74 Ma (95% highest posterior density (HPD): 35.73-39.87) in the later of Eocene and 15.68 Ma (95% HPD: 6.7-26.65) in the middle Miocene, respectively (**Figure 6**). Phylogenetic inference of plastome haplotypes subdivided 12 haplotypes into five main clades. Molecular dating analysis suggested that the firstly diverged during the later Miocene, 8.42 Ma (95% HPD: 3.48-14.97). The second divergence was occurred in the 4.82 Ma (95% HPD: 2.08-8.16) in the middle of Pliocene, giving the clade V. The crown age of Clade II, Clade III, and Clade IV and the divergence time between Clade II and Clade III were also in the middle of Pliocene. The divergence time of different genotypes within the clade was occurred in the Pleistocene.

## Discussion

### The plastome of *Pistacia chinensis* is highly variable

With the advent of next generation sequencing, plastome sequence data has become a basic tools and is extensively used to resolve evolutionary relationships among plant species at different taxonomic levels (Sloan et al., 2014; Dong et al., 2018; Wikström et al., 2020; Zhao et al., 2021; Dong et al., 2021a; Dong et al., 2021c). Moreover, the capacity to assess the mutation rate or detect variation has significantly improved. Many studies have focused on variations at the species level or above (Liu et al., 2021; Ren et al., 2021; Tian et al., 2021; Xiong et al., 2021), however, only few studies have examined intraspecific diversity for whole plastome sequences (Hu et al., 2022; Lian et al., 2022; Sun et al., 2022). Plastid markers have been used in tree population genetics but were often limited to few polymorphic sites (Mariotti et al., 2010; Huang et al., 2014; Mohamoud et al., 2019). In this study, we sequenced the plastomes of 39 individuals of *Pistacia chinensis* sampled from the germplasm bank, and assessed the variation in the genomes. In total, 460 intraspecific polymorphic sites, 104 indels and three small inversions were identified among these 39 individuals. Mutation diversity of the plastome in *P. chinensis* is relatively high compared to that published for other plant species, including *Arnebia guttata* (313 polymorphic sites, 17 individuals) (Sun et al., 2022), *Bretschneidera sinensis* (105 polymorphic sites, 55 indels, 12 individuals) (Shang et al., 2022), the model grass plant *Brachypodium distachyon* (298 polymorphic sites, 53 individuals) (Sancho et al., 2018), and *Ginkgo biloba* (135 polymorphic sites, 71 individuals) (Hohmann et al., 2018).

Mutation rate variation among different lineages of plastomes has been examined in various studies (Smith and Donoghue, 2008; Schwarz et al., 2017; Choi et al., 2021). A hypothesis commonly invoked is that mutation rates are negatively correlated with generation time (Dong et al., 2022). For example, long-lived woody plants have lower mutation rates that do short-lived herbaceous species (Smith and Donoghue, 2008; Amanda et al., 2017; Dong et al., 2022), and the reed canary grass *Phalaris arundinacea* has high intraspecific plastid diversity (Perdereau et al., 2017). However, the relatively high intraspecific mutation rate we see in *Pistacia chinensis*, a long-lived plant, is not satisfactorily explained by the generation time hypothesis. Divergence time estimation indicated that *P. chinensis* speciated very early, during the late Miocene, 8.42 Ma (**Figure 6**). Relatively high genetic diversity is therefore likely to be a consequence of persistence of genetically distinct populations through periods of historical climate variability during the long evolutionary history. From the Pliocene to the Early Pleistocene, Eastern China was affected by the uplift of the northeastern and Southeastern Tibet Plateau (An et al., 2006), and the intensification of the East Asian summer monsoon (EASM) (An et al., 2014) and South Asian summer monsoon (SASM) (Chang et al., 2010) also occurred during this time.

Regions of the plastome with higher mutation rates (so-called mutational hotspot regions) have been observed in previous research, and the IR regions are known to be more conserved than the LSC and SSC regions (Dong et al., 2021b; Dong et al., 2021c). In the *P. chinensis* plastome, intraspecific variable sites and indels were mostly located in the LSC and SSC regions. Regions of particularly high variability in *P. chinensis* included *trnH-psbA* and *ndhF-rpl32*. Both have been identified as universal and variable markers suitable for intraspecific level studies, such as investigations into genetic diversity or population structure.

### Genetic diversity in *Pistacia chinensis*

Using plastid and nuclear genome data, we provided much needed information on the genetic diversity of *P. chinensis.* Population structure results revealed five and two clusters of *P. chinensis* with high levels of diversity, using the plastid and nuclear genomes, respectively (**Figures 2** and **4**). High genetic differentiation was detected between distant clusters, however, these clusters did not reflect geography, suggesting that geographic distance did not explain the patterns in genetic structure according to the plastome dataset. For example, for the Clade I in the plastome dataset, this clade included the samples from nine provinces of China. The Clade III included four samples, which located in the four separate provinces of China (Guangxi, Jiangxi, Jiangsu, and Yunnan) (**Figure 2B**).The SSR results found similar results for the eight sampled *P. chinensis* populations, with different populations showing high differentiation and some of the distant populations showing high genetic similarity (Wu et al., 2010). Other factors may therefore be driving the population structure of *P. chinensis*, such as gene flow or introgression. When exclude the samples with hybrids, the samples in the stu01 group were most located in the east of China, and the stu02 group were most located in the west of China (**Figure 5**). This indicated both clusters reflected the geography excluding the effective of gene flow or introgression.

Most individuals at the tips of the phylogeny and population structure displayed apparent discordance between their nuclear and plastomes. This can be caused by factors such as hybridization and/or introgression between highly divergent populations (Wang et al., 2019; Cui et al., 2020). Most of the individuals in the *P. chinensis* subclade diverged between the late Miocene and the mid-Pliocene. The southern Chinese distribution of *P. chinensis* may have been subject to climate change and intensification of the EASM and SASM, creating opportunities for mixing and introgression between the early divergent clades.

On top of the evolutionary factors, cultivation may be a major factor effecting the genetic diversity of *P. chinensis*. Genetic diversity is generally thought to decrease with cultivation (Varshney et al., 2021; Wei and Jiang, 2021), as not all genotypes will be retained. Meanwhile, hybridization under artificial intervention or natural hybridization further leads to the mixing of wild resources. High heterozygosity is one characteristic of many cultivated plants and one of many recognized challenges facing plant breeding. For *P. chinensis*, one third of the sampled individuals were identified as hybrids. Clade I from the plastid data included 25 samples from Shandong, Beijing, Shanxi, Jiangsu, Zhejiang, Gansu, Guizhou, Hebei, and Anhui. The plastome sequences of these samples were very similar to each other, suggesting that this clade may comprise the cultivated individuals. Our results indicated that during the cultivation process, the genetic diversity of *P. chinensis* may have decreased, suggesting that more genotypes, which are potentially of use in further breeding of this important species, should be conserved.

## Data availability statement

The datasets presented in this study can be found in online repositories. The names of the repository/repositories and accession number(s) can be found below: https://www.ncbi.nlm.nih.gov/genbank/, OP554519- OP554557.

## Author contributions

BH, X-MX and W-QL conceived and designed the study. BH, M-JZ, YX, C-CC, HX and DL collected and analyzed the data. BH wrote the manuscript. LW edited and improved the manuscript. All authors contributed to the article and approved the submitted version.

## Funding

This study was supported by the Project funded by the Postdoctoral Science Foundation “Research and development of key technologies and equipment of germplasm bank” (BSHCX202101), the Postdoctoral Station Recruitment Subsidy of Shandong Province “Collection, preservation, evaluation and utilization of *Quercus acutissima* and *Q. variabilis* Germplasm Resources” (BSHCX202102).

## Acknowledgments

We appreciate the facilitation provided by National Wild Plant Germplasm Resource Center. And we thank Dr. Jane Marczewski for polishing the English text professionally.

## Conflict of interest

The authors declare that the research was conducted in the absence of any commercial or financial relationships that could be construed as a potential conflict of interest.

## Publisher’s note

All claims expressed in this article are solely those of the authors and do not necessarily represent those of their affiliated organizations, or those of the publisher, the editors and the reviewers. Any product that may be evaluated in this article, or claim that may be made by its manufacturer, is not guaranteed or endorsed by the publisher.

## Supplementary material

Supplementary Table 1 The haplotypes of 39 *Pistacia chinensis* samples.

Supplementary Figure 1 Gene maps of the *Pistacia chinensis* plastome. The genes are color-coded based on their functions. The dashed area indicates the GC composition of the plastome.

Supplementary Figure 2 The results of CV error for the plastome and SNPs datasets.

Supplementary Figure 3 Principal component analysis based on the nuclear SNPs dataset.
