# Supplementary figures and images for: Variations in genetic diversity in cultivated Pistacia chinensis
Source: Front Plant Sci. 2022 Nov 10;13:1030647. doi: 10.3389/fpls.2022.1030647 (PMC9691265; doi:10.3389/fpls.2022.1030647)

a. Plastome

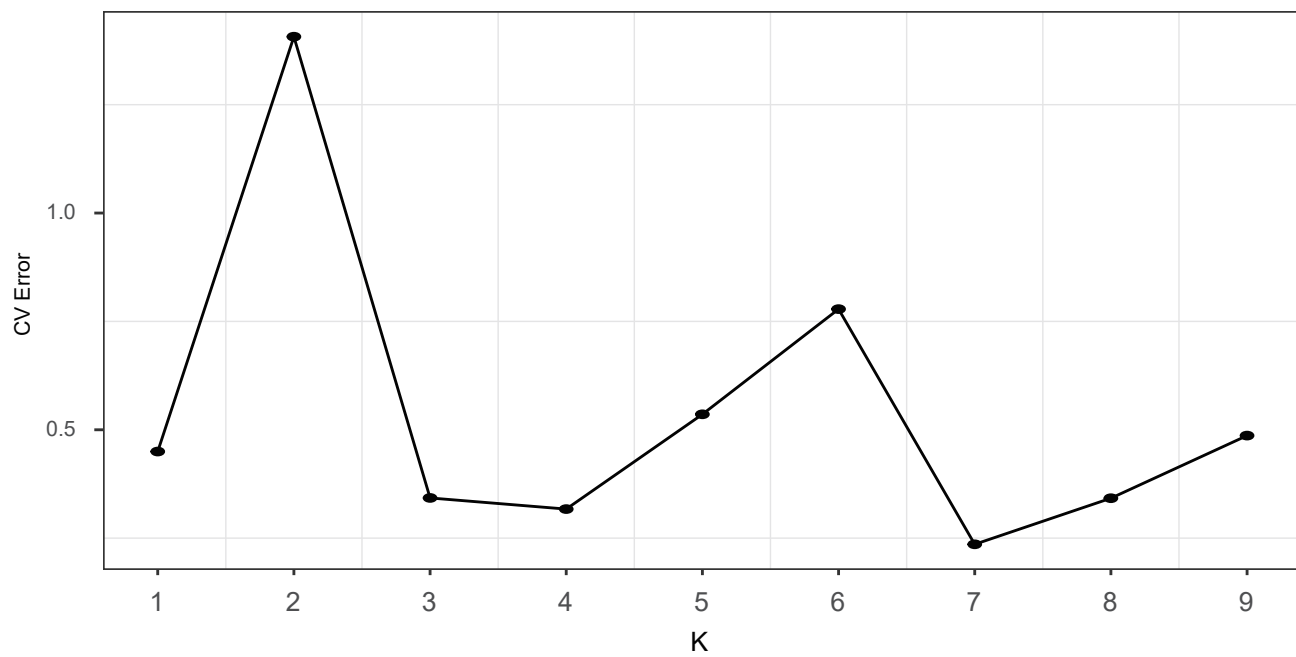

b. SNP

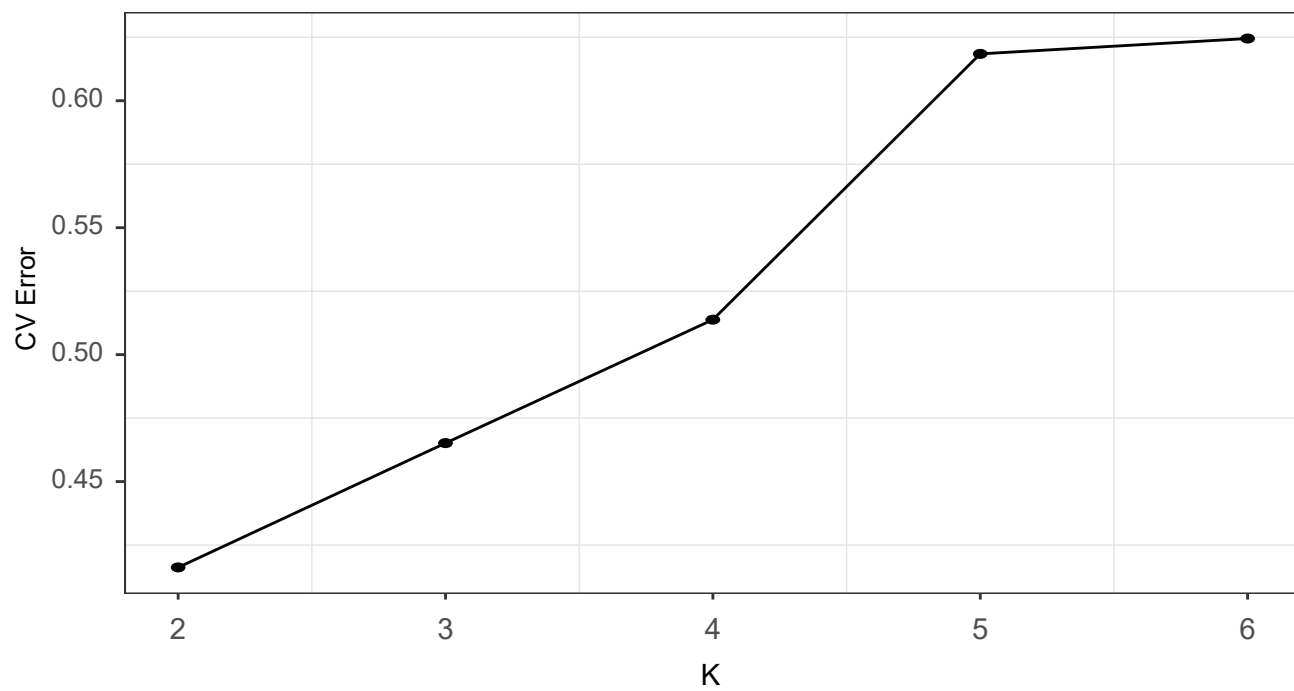

Supplement: Supplementary Figure 2 — The results of CV error for the plastome and SNPs datasets. [file DataSheet_2.pdf]

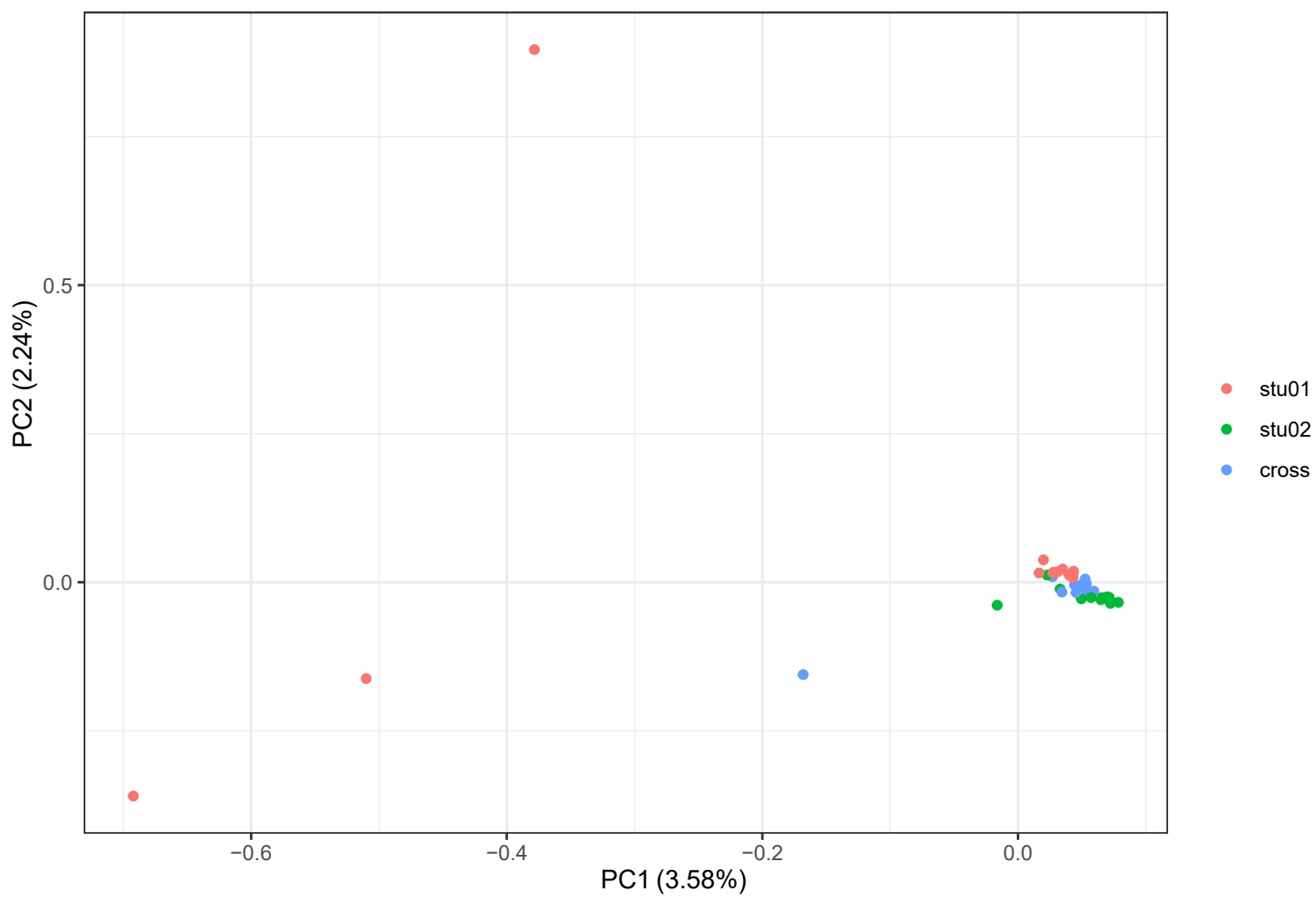

Supplement: Supplementary Figure 3 — Principal component analysis based on the nuclear SNPs dataset. [file DataSheet_3.pdf]
